# The Modified Superomedial Pedicle Breast Reduction Technique for Cases with SN–N Distance Exceeding 33 cm

**DOI:** 10.1007/s00266-024-04174-z

**Published:** 2024-06-21

**Authors:** Georgina Panopoulou, Ilias Petrou, Demetris Savva, Andreas Vassiliou

**Affiliations:** 1 Plastic and Reconstructive Surgery Department, Nicosia General Hospital, 2031 Nicosia, Cyprus; 2 School of Medicine, European University Cyprus, 2404 Nicosia, Cyprus; 3 School of Pharmaceutical Sciences and Health Products, University of Camerino, 62032 Camerino, Italy

**Keywords:** Modified superomedial pedicle, Breast reduction, Reduction mammaplasty, Breast hypertrophy, Macromastia, Gigantomastia

## Abstract

**Background:**

Symptomatic breast hypertrophy affects the quality of life of a large number of women globally. Many reduction mammoplasty techniques have been described for patients with breast hypertrophy. The aim of this study was to provide our clinic’s experience in utilizing the modified superomedial pedicle breast reduction technique in specific patients suffering from breast hypertrophy, with sternal notch-to-nipple distance of more than 33 cm.

**Method:**

Our study included twenty patients who underwent, from January 2022 to December 2023, the modified superomedial pedicle breast reduction technique due to symptomatic breast hypertrophy with sternal notch-to-nipple distance of more than 33 cm in the Plastic and Reconstructive Surgery Department at Nicosia General Hospital in Cyprus. Patient demographics, comorbidities, pre- and postoperative breast anthropometric measurements and surgical complications were recorded and analyzed.

**Results:**

The average age at the time of the reduction was 48 years. The mean preoperative body mass index was 28.52 kg/m^2^. Patients’ comorbidities included one (5%) patient with diabetes, seven (35%) with obesity and three (15%) with hypertension. The mean preoperative sternal notch-to-nipple distance was 35.25 cm for the right breast and 34.90 cm for the left breast, while the mean postoperative was 20.65 cm for both breasts. The total mean resection weight of both breasts was 1643.45 g. Surgical complications were minor including two (10%) cases of local hematoma and one (5%) case of T-Junction wound breakdown. All patients were relieved from their preoperative symptoms and were satisfied with the final result.

**Conclusion:**

Our modified superomedial pedicle technique is a safe, effective and versatile pedicle to be used with many advantages, in specific patients suffering from breast hypertrophy with sternal notch-to-nipple distance of more than 33 cm, including its shape and rotational abilities, viability of the nipple and excellent outcome of glandular plication and breast reshaping.

**Level of Evidence IV:**

This journal requires that authors assign a level of evidence to each article. For a full description of these Evidence-Based Medicine ratings, please refer to the Table of Contents or the online Instructions to Authors www.springer.com/00266.

**Supplementary Information:**

The online version contains supplementary material available at 10.1007/s00266-024-04174-z.

## Introduction

As stated by 2022 International Society of Aesthetic Plastic Surgery statistics (ISAPS), the operation of breast reduction is the tenth most performed procedure by Plastic Surgeons on a global scale [1]. It is widely known that symptomatic breast hypertrophy affects the quality of life of a large number of women globally [2].

Breast hypertrophy serves as an overarching term encompassing both macromastia and gigantomastia, which represent varying degrees of excessive breast tissue growth, each resulting in distinct clinical implications [3–5]. Gigantomastia is broadly defined as a condition necessitating the removal of more than 2500 g of breast tissue, while macromastia is defined as a condition necessitating the removal of less than 2500 g of breast tissue [6, 7]. However, the exact designation of breast hypertrophy remains controversial due to differing guidelines. For the purposes of our study, breast hypertrophy is arbitrarily defined as a bilateral breasts resection tissue weight estimation amount of 2000 g, in patients with sternal notch-to-nipple (SN–N) distance of more than 33 cm.

Another definition of breast hypertrophy is the Sacchini criteria which are based on the nipple to the inframammary fold distance (N–IMF) and the nipple to the lateral sternal margin [8]. Three categories were identified: Distance less than 9 cm indicates a small size breast; between 9 and 11 cm indicates a medium size breast; and lastly greater than 11 cm indicates a large size breast [8]. Additionally, some studies use the bra size as a criterion of measurement. For example, a cup D size or larger is considered a hypertrophied breast [9]. Insurance companies in the USA rely upon the Schnur Sliding Scale to determine patient eligibility for breast reduction surgery. The patient’s body surface area (BSA) and average weight of breast tissue removed are incorporated into this scale in order to identify women necessitating breast reduction for medical reasons [10].

Women with breast hypertrophy often experience a spectrum of symptoms. According to a systematic review, the most frequent symptomatology is shoulder grooving, followed by shoulder, neck pain and intertriginous infections [11]. Furthermore, some patients complain of headaches, numbness in upper extremities and in extreme cases they present with degenerative joint disease of the cervical or thoracic spine, which can significantly impact their physical and psychological well-being [11–13]. Standard conservative approaches such as customized brassieres, weight management and physical therapy are frequently recommended [14, 15]. However, these methods sometimes fall short in adequately addressing symptoms and satisfying patients [14–16]. In such cases where non-surgical interventions prove ineffective, reduction mammoplasty is then become a viable option.

The objective of this study is to provide our clinic’s experience in utilizing the modified superomedial pedicle breast reduction technique for a specific category of patients suffering from breast hypertrophy. We aim to demonstrate its safety and effectiveness in alleviating symptomatic patients and preserving the nipple–areolar complex (NAC) while achieving optimal aesthetic breast results in cases where the SN–N distance is greater than 33 cm.

## Materials and Methods

### Patients

Our study, conducted from January 2022 to December 2023 in the Plastic, Reconstructive and Aesthetic Surgery Department at Nicosia General Hospital, includes 20 patients that underwent, for the first time, the bilateral modified superomedial pedicle breast reduction technique. Inclusion criteria were limited to symptomatic breast hypertrophy greater than 1 year: headache, shoulder grooving, chronic breast/neck/back pain, intertriginous infections and lastly emotional sequelae particularly in young girls. Additionally, selection criteria included SN–N distance of more than 33 cm and a bilateral breasts resection tissue weight estimation amount of 2000 g. Patients who underwent unilateral breast reduction, oncoplastic breast reduction and any kind of bariatric surgery were excluded from this study. In the exclusion criteria were also patients with high malignant breast potential as well as breast cancer patients. Operation was postponed for patients who were planning a pregnancy and lose weight. All patients were treated by the same attending plastic surgeon and his team and followed the same postsurgical care for the purpose of this study.

The age, weight and height of every patient were written down, and the body mass index (BMI) was calculated. Patients’ smoking status and comorbidities like obesity, hypertension and diabetes were also obtained from patients’ medical history. In addition, breast anthropometric measurements were recorded both pre- and postoperatively, such as the SN–N distance, the N–IMF and the breast base width (BBW). During the operation, each excised breast tissue was weighted and recorded. Last but not least, the complications occurred were minor. Patients were monitored regularly at the hospital’s outpatient breast clinic, for a period of not less than 6 months.

*Ethical Considerations and Patient’s Consent*

All procedures performed in this study were in accordance with the ethical standards of the institutional and/or national research committee and with the 1964 Helsinki Declaration and its later amendments or comparable ethical standards. After explaining the purpose and the nature of the study, informed consent was obtained and signed from the subjects who accepted to participate. Anonymity and confidentiality of participants were ensured by the authors.

### Dara Collection and Statistical Analysis

Patients’ data collection and basic statistical analysis were initiated using Airtable platform and Microsoft (MS) Excel sheets. Descriptive statistics (mean ± SD) were employed to express continuous variables, while frequencies and percentages were used for categorical variables.

## Management and Surgical Technique

### Preoperative Phase

Detailed medical history was obtained from patients during the preoperative phase. Comprehensive explanation of the operative procedure and realistic expectations regarding breast outcomes was thoroughly discussed and ensured. Patients were fully informed about potential risks and complications associated with the surgery, as well as its benefits. They were also informed that surgical procedure will be carried out under general anesthesia. Breasts examinations were conducted, and photographs were taken for medical records. Our protocol was filled with precise breast anthropometric measurements and completed in subsequent outpatient visits (see Appendix 1). To ensure optimal surgical planning, we mandated routine laboratory workup, a mammogram, a minimum of 6 weeks smoking cessation before surgery and temporary discontinuation of aspirin and anti-inflammatory drugs.

**Fig. 1:**
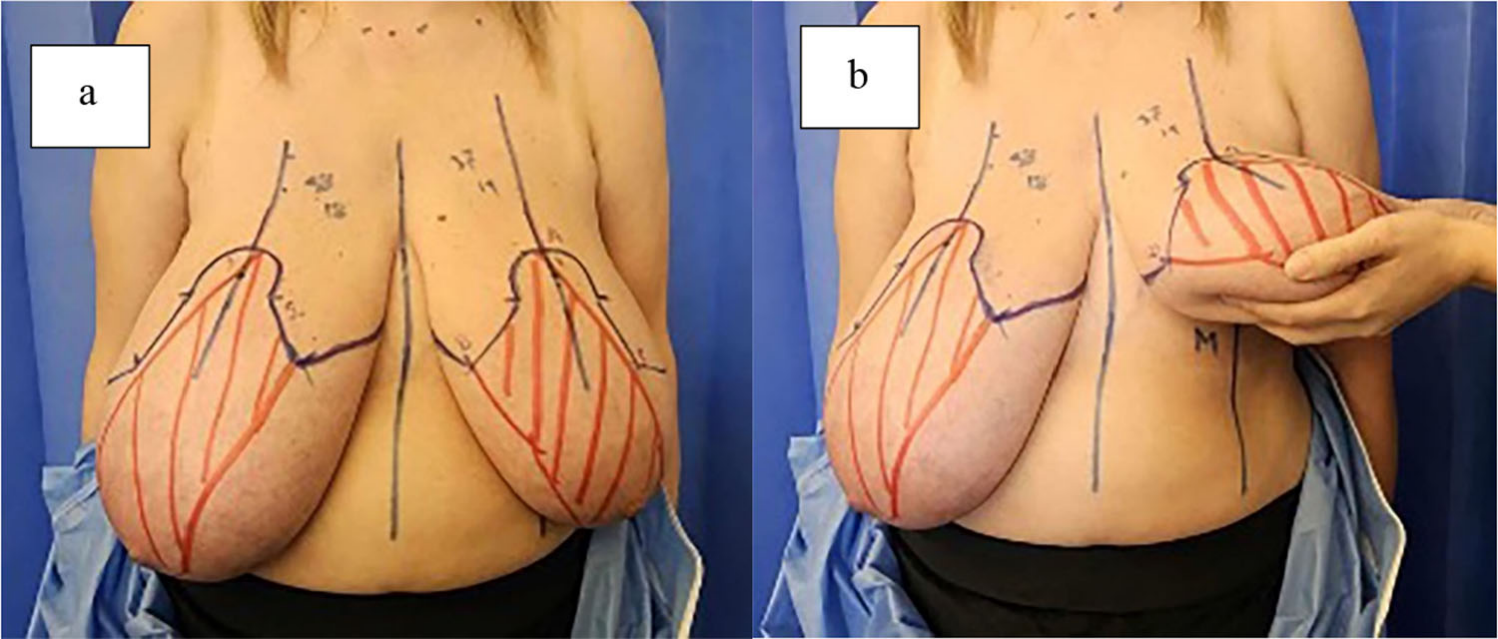
Preoperative view of a 53-year-old woman with breast hypertrophy. **a** Patient is marked in the upright position. **b** Folding up the breast to find the upper breast border. Superomedial pedicle is shown with red color forming a U shape. M: breast Meridian

On surgery day, as part of the skin preparation prior to the surgery, all patients were instructed to douche using a 7.5% povidone–iodine surgical scrub solution. Preoperatively, patients were marked in the upright position. The sternal notch, inframammary fold, breast meridian and chest midline are drawn (Fig. 1a). The upper breast border (UBB) is marked by folding up the breast; the UBB is at the junction of the pre-axillary fold and the breast at the level of the indentation (Fig. 1b). The new nipple–areolar complex (NAC) position is determined with transposition of the inframammary crease onto the anterior surface of the breast, usually put at 18–22 cm. The areola is usually planned at a 3.8 cm diameter. The superomedial pedicle on a Wise-pattern template is marked with a different color. The vertical and lateral limbs are marked according to the amount of skin needed to be removed [17, 18].

### Operative Phase

**Fig. 2:**
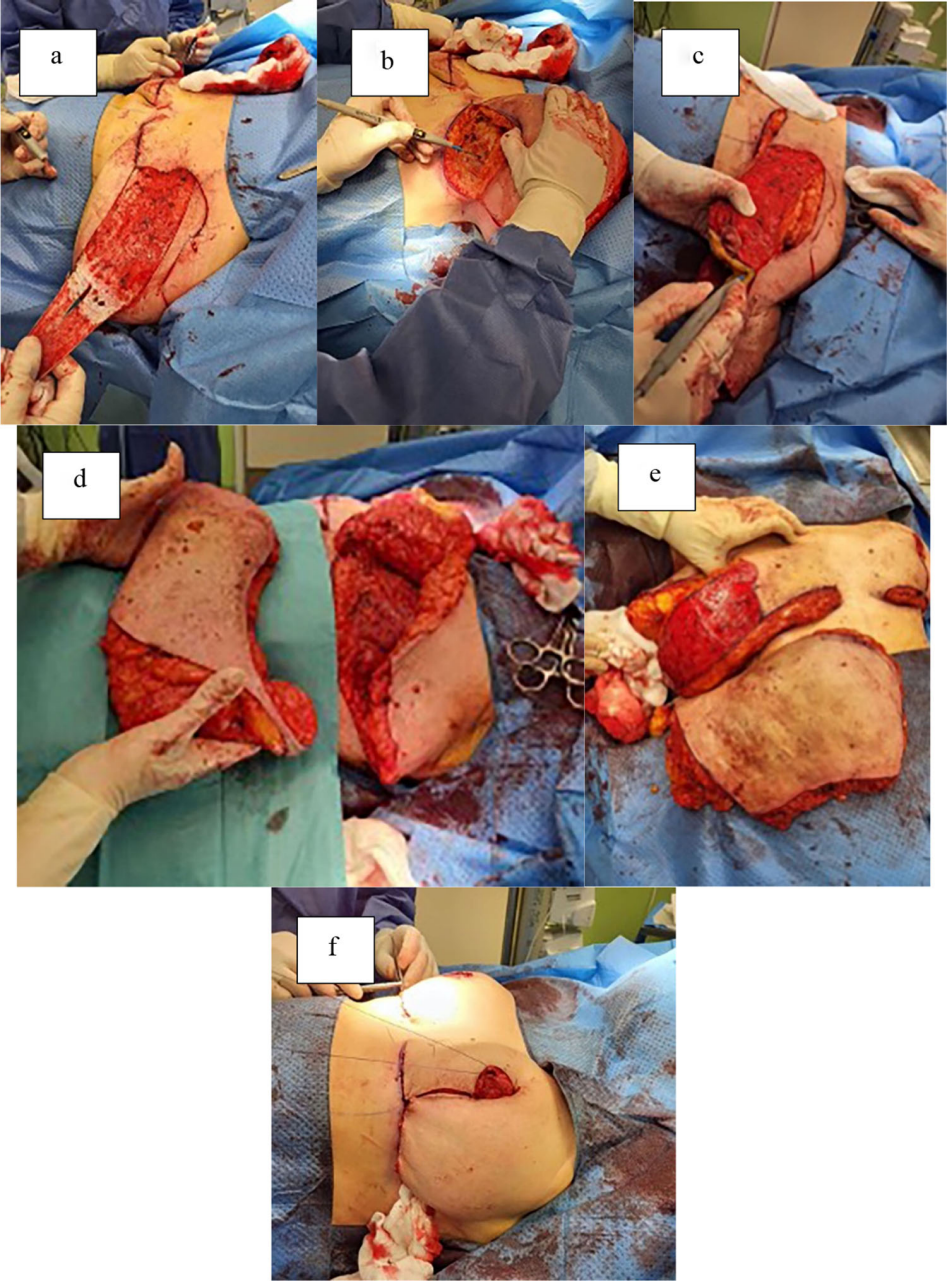
Surgical technique. **a** Deepithelization of the pedicle. **b** The base of the breast dissected off the underlying chest wall muscle fascia. **c** Performing the lower breast parenchymal excision. **d** Tissue resected from the left breast. **e** Tissue resected from the right breast. **f** The pedicle is rotated and temporarily closed

During operation, the patients were positioned supine with their arms abducted on the operating room table. A cookie cutter is used to mark the new desired NAC circumference (diameter approximately 3.4 cm) [17, 18]. The operation starts with de-epithelialization of the pedicle (Fig. 2a), preserving the NAC and then undermining the whole upper portion of by the inframammary fold (IMF) incision down to the pectoralis fascia [17] (Fig. 2b). Excision of the extra dermoglandular tissue on the lateral and inferior aspects of the pedicle is then performed according to previous markings as well as intraoperative decision (Fig. 2c–e). Afterward, the pedicle is rotated by anchoring the NAC at its outer limit. This rotation follows a clockwise approach: from three o’clock toward the breast midline/meridian for the left breast and from nine o’clock toward the breast midline/meridian for the right breast. Additionally, a wedge excision is performed on the pillow adjacent to the flap pivot point. Then the pedicle, with maximum pedicle width-to-length ratio of 2:1, is inset on the upper pole of the breast. Some adjustments to size and limb length can be made at this point. Additionally, surgical drains are placed in cases where more than 1000 g of bilateral breast tissue is removed. The incisions are closed in a layered fashion with 2-0 PDS and 3-0 Monocryl interrupted sutures (Fig. 2f). Finally, running 3-0 Monocryl subcuticular sutures are placed. The breasts are then dressed with a wide fixing dressing tape, and a medical bra is placed. A standardized chemotherapeutic protocol was adhered to for all patients, consisting of one perioperative dose and two postoperative doses of second-generation cephalosporin. Analgesia was administered according to patients’ needs.

### Postoperative Phase/Follow-Up

Following surgery, patients were instructed to wear a compression bra daily, with the exception of shower time, for a minimum of 2 weeks. Subsequently, a transition bra was recommended again for daily wear for an additional time period of 4 weeks. After 6 weeks, patients were advised to wear any bra without an underwire. Long-term bra usage, 3 months postoperative, was tailored based on individual’s surgical incision healing progress. Showering was initiated 3 days after surgery, and non-absorbable sutures were removed 2 weeks postoperative. Social activities, as well as driving, were recommended 1 week postoperative. Surgical dressing changes were scheduled at intervals of three, seven and 14 days, while follow-up visits were initiated at intervals of three, seven, fourteen, thirty, ninety and lastly 180 days.

## Results

A total number of 20 patients suffering from breast hypertrophy underwent for the first time, between January 2022 and December 2023, the bilateral modified superomedial pedicle breast reduction technique. The patients’ mean age was 47.95 ± 13.79 years (range 23–69 years), mean preoperative weight was 74.20 ± 11.29 kg (range 58–103 kg), and mean height was 1.61 ± 5.38 cm tall (range 1.53–1.73 cm tall). Furthermore, the mean preoperative body mass index (BMI) was 28.52 ± 5.38 kg/m^2^ (range 20.38–44.00 kg/m^2^). Patients’ smoking status and comorbidities are illustrated in Table 1.

**Table 1:** Patients’ smoking status and comorbidities

Smoking status	N (%)
Never	15 (75)
Former	4 (20)
Current	1 (5)
Comorbidities	N (%)
Diabetics	1 (5)
Obesity (BMI ≥ 30 kg/m^2^)	7 (35)
Hypertension	3 (15)

The mean right breast excised weight was 849.80 ± 478.05 g (range 160–2330 g), and the mean left breast excised weight was 794.10 ± 483.36 g (range 150–2130 g). The total mean resection weight of both breasts was 1643.45 ± 950.65 g (range 310–4460 g). Patients’ breast anthropometric measurements pre- and postoperatively are illustrated in Table 2.

**Table 2:** Patients’ breast anthropometric measurements

	SN–N	N–IMF	BBW
*Right Breast*			
Range preoperative (cm)	33–43	9–22	12–19
Mean preoperative (cm)	35.25 ± 2.89	14.70 ± 3.46	16.55 ± 1.79
Range postoperative (cm)	17–27	8–18	5–19
Mean postoperative (cm)	20.65 ± 2.45	9.30 ± 2.63	13.95 ± 3.34
*Left Breast*			
Range preoperative (cm)	33–45	10–22	13–19
Mean preoperative (cm)	34.90 ± 3.24	14.95 ± 3.69	16.60 ± 1.66
Range postoperative (cm)	17–27	8–18	5–19
Mean postoperative (cm)	20.65 ± 2.45	9.25 ± 2.67	13.95 ± 3.34

**Fig. 3:**
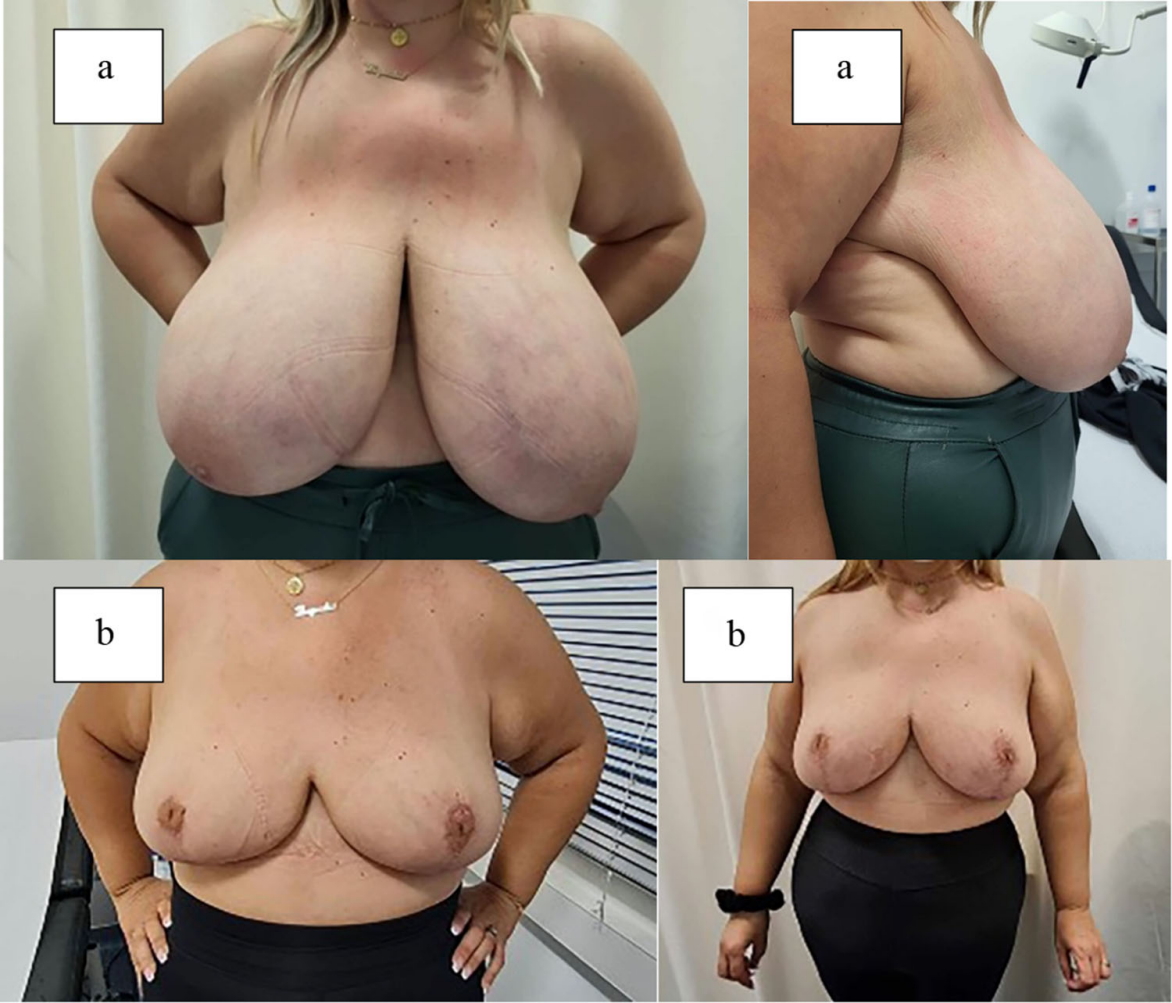
Patient before and after surgery. **a** Patient presented with initial sternal notch–nipple distance of 43 cm on the right and 45 cm on the left side with a nipple–inframammary fold of 18 cm on the right and 22 cm on the left side. **b** Long-term follow-up at 6 months after removal of 1500 g on the right and 1800 g on the left breast

**Fig. 4:**
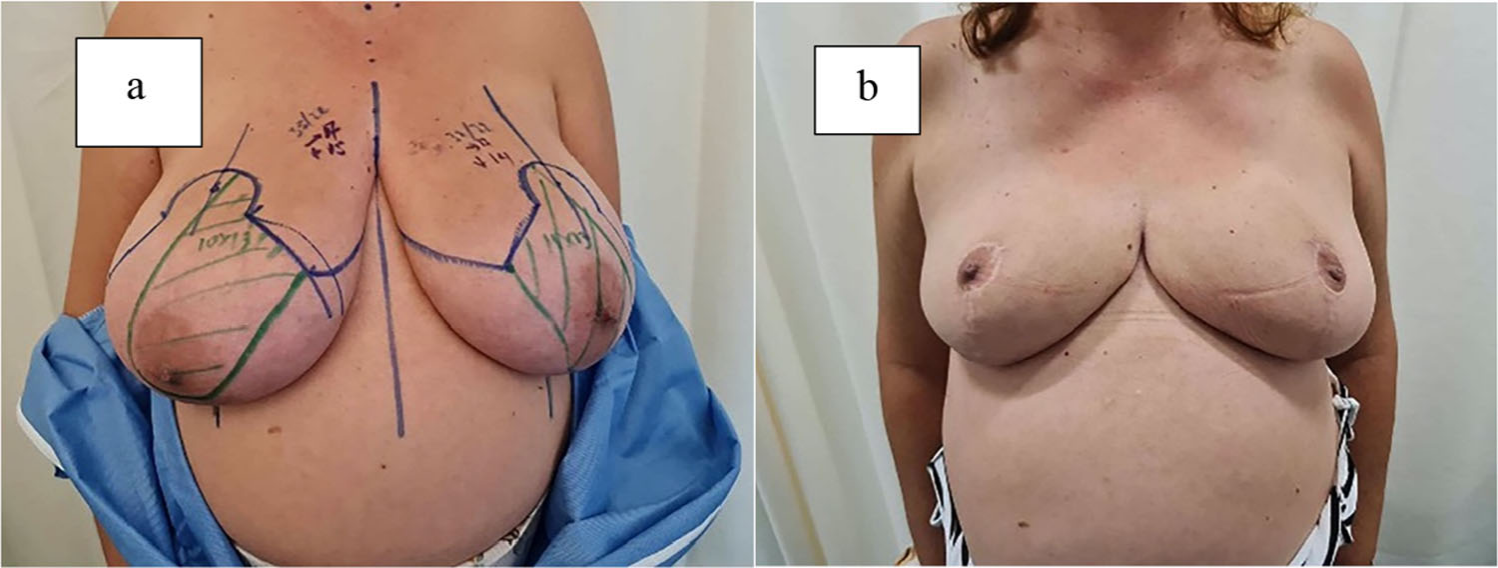
Patient before and after surgery. **a** Patient presented with initial sternal notch–nipple distance of 35 cm on the right and 33 cm on the left side with a nipple–inframammary fold of 15 cm on the right and 14 cm on the left side. **b** Long-term follow-up at 6 months after removal of 1090 g on the right and 880 g on the left breast

Postoperative complications were minor including two (10%) cases of local hematoma and one (5%) case of T-Junction wound breakdown. Upon leaving the hospital, patients were monitored in the outpatient clinic of our department at regular intervals and according to need (see postoperative phase/follow-up). Figures 3 and 4 show patients preoperatively and at 6 months postoperatively.

## Discussion

There is a broad diversity of techniques for breast reduction. Various combinations of incisions and pedicles have been outlined to achieve the objectives of reduction mammoplasty, which include: (a) preserving the vascularity and innervation to the NAC, (b) achieving optimal volume reduction, (c) forming a skin envelop that minimizes scarring and maintains proportionality with the remaining breast tissue and lastly (d) sustaining a favorable aesthetic breast shape [15]. These criteria are met with the utilization of our modified superomedial pedicle breast reduction technique.

In this study, the calculated mean age of participants was 47.95 years, comparable to findings from other studies [4, 19]. Furthermore, patients’ mean preoperative body mass index (BMI) was 28.52 kg/m^2^, consistent with the results of Grover et al. [20], who reported a mean perioperative BMI of 29.6 kg/m^2^. In our study, the mean right breast excised weight was 849.80 g and the mean left breast excised weight was 794.10 g. These findings align closely with the results of the two studies conducted by Lugo et al. and Toplu et al. [12, 19], demonstrating an average breast excised weight of 1277 g for the right and 1283 g for the left breast, and 1100 g for the right and 1090 g for the left breast, respectively.

Regarding patients’ breast anthropometric measurements, the mean preoperative SN–N distance was measured at 35.25 cm for the right breast and at 34.90 cm for the left breast. These findings were supported again by Lugo et al. and Toplu et al. [12, 19], who reported a mean SN–N distance of 35.5 cm for the right breast and 35.6 cm for the left breast, and 27 cm for the right and 26 cm for the left breast, respectively. Additionally, in our study, the mean postoperative SN–N distance was measured at 20.65 cm for both breasts, an almost identical finding to the study conducted by Toplu et al [19], demonstrating a mean postoperative SN–N distance of 20.5 cm.

Reduction mammoplasty has proven to be an effective intervention for patients suffering from breast hypertrophy by all accounts [3, 11, 12]. However, it is associated with complications that occur with varying frequencies in the literature [12, 18, 20]. In our study, postoperative complications were minor including two (10%) cases of local hematoma and one (5%) case of T-Junction wound breakdown observed. These findings are comparable to other studies [18, 20].

The aim of our study was to evaluate the use of the modified superomedial pedicle breast reduction in cases of breast hypertrophy with SN–NAC distance of > 33 cm, while preserving the nipple–areolar complex (NAC) and achieving at the same time optimal aesthetic breast results. Incessantly, the SMP has been popularized for reduction mammoplasties as it is considered a safe and effective pedicle to use, especially in big volume resections of up to 2000 g and NAC transpositions of up to 15 cm [21]. What’s more, a better cosmetic durability (less bottoming out or pseudoptosis over time), reduced operative time and better appearance (fuller medial breast volumes and cleavage) are observed with the use of the SMP as the vascular pedicle for breast reduction.

## Addressing Limitations

While our study provides valuable insights into the use of modified superomedial pedicle breast reduction technique for a selective category patients, it is important to acknowledge some limitations. Regarding the subjectivity of results, we acknowledge that the interpretation of qualitative data can be influenced by subjective factors. To address this concern, we have taken several steps to enhance the rigor and reliability of our analysis. Strategies to address subjectivity in our study included: (a) transparent methodology, (b) multiple observers, (c) standardization and lastly (d) reflexivity. Furthermore, we recognize the absence of control groups as a limitation of our study. While control groups are commonly used in experimental research to establish causal relationships, our research employed an observational study, which limits our ability to control for confounding variables. Additionally, the mean follow-up time was 6 months; however, it is important to recognize that this duration may not provide a comprehensive representation of long-term outcomes. Despite these limitations, we believe that our study design allowed us to capture real-world clinical experiences, which are valuable for establishing best practices in clinical settings for other colleagues.

## Conclusion

Summarily, the modified superomedial pedicle technique is a safe and effective approach utilized for patients with sternal notch-to-nipple distance of more than 33 cm. It offers numerous advantages, including its shape and rotational capabilities, preservation of nipple viability and excellent outcome of glandular plication and breast reshaping.

## Supplementary Information

Below is the link to the electronic supplementary material.

Supplementary file1 (DOCX 244 KB)

## Data Availability

The data used and analyzed during the current study are available from the corresponding author on reasonable request.
