# Supplementary material for: The Modified Superomedial Pedicle Breast Reduction Technique for Cases with SN–N Distance Exceeding 33 cm
Source: Aesthetic Plast Surg. 2024 Jun 21;49(1):139–46. doi: 10.1007/s00266-024-04174-z (PMC11799024; doi:10.1007/s00266-024-04174-z)
Supplement: Supplementary file 1 — Supplementary file1 (DOCX 244 KB) [file 266_2024_4174_MOESM1_ESM.docx]

**Breast Protocol**

**Phone number: _____________________**

**Responsible doctor: _____________________**

**Diagnosis: _________________________________________________________________**

**Reason of entry: ____________________________________________________________**

**Type of surgery: ____________________________________________________________**

| **Personal history:** | **Surgical history:** | **Drug history:** |
| --- | --- | --- |
|  |  |  |
|  |  |  |
|  |  |  |
|  |  |  |
|  |  |  |
|  |  |  |
|  |  |  |

**Actinotherapy: _____________________________________________________________**

**Chemotherapy: _____________________________________________________________**

**Ormonotherapy: ____________________________________________________________**

**Allergies: __________________________________________________________________**

**Smoking: __________________________________________________________________**

**Alcohol: ___________________________________________________________________**

**Height: _____________________**

**Weight: _____________________**

**BMI: ________________________**

**Physical examination: _________________________________________________________________________________________________________________________________________________________________________________________________________________________________**

**
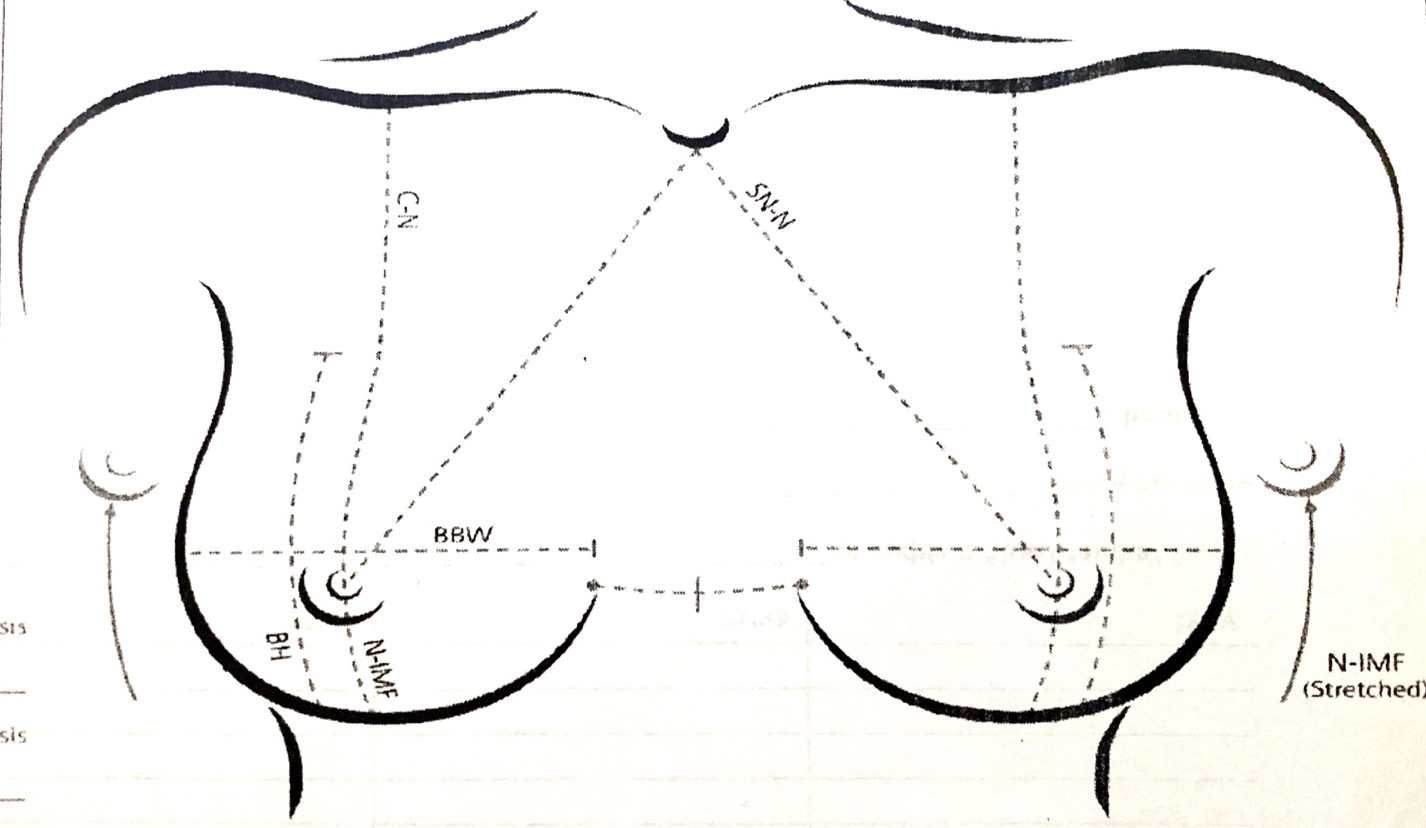
**

|  | **Postoperative** | | **Preoperative** | | **6 months** | |
| --- | --- | --- | --- | --- | --- | --- |
|  | **LT** | **RT** | **LT** | **RT** | **LT** | **RT** |
| **SN-N** |  |  |  |  |  |  |
| **N-IMF** |  |  |  |  |  |  |
| **BBW** |  |  |  |  |  |  |

**Type of surgery:**

- Augmentation
- Mastopexy
- Reduction
- Reconstruction
- Augmentation mastopexy
- Revision

**Incision:**

- IMF
- Periareolar
- Vertical
- Wise Pattern
- Axillary
- Other: **_________________**

**Flap type:**

- Superior
- Inferior
- Superomedial
- McKissock
- Other: **_________________**

**IMF location:**

- Preserve
- Elevation
- Lower

**Implant placement:**

- Subpectoral
- Subfascial
- Subglandular

| **Breast implants** |
| --- |
| **LT** |
| **RT** |
